# Model for Taking Care of Patients with Early Childhood Caries during the SARS-Cov-2 Pandemic

**DOI:** 10.3390/ijerph17113751

**Published:** 2020-05-26

**Authors:** Stefano Cianetti, Stefano Pagano, Michele Nardone, Guido Lombardo

**Affiliations:** 1 Biomedical Sciences, Unit of Paediatric Dentistry, University of Perugia, 06100 Perugia, Italy; stefano.cianetti@unipg.it (S.C.); guido.lombardo@unipg.it (G.L.); 2 Azienda Socio-Sanitaria Territoriale, Melegnano e della Martesana, 20070 Milano, Regione Lombardia, Italy; logu68@libero.it

**Keywords:** SARS-CoV-2, early childhood caries, minimally invasive, infection control

## Abstract

Pending the availability of vaccines to contain the SARS-CoV-2 pandemic, the current solution is “social distancing” with a reduction of dental treatments to those assessed as urgent and emergency cases. These treatments also involve Early Childhood Caries (ECC) due to the fact that this disease affects preschool children (a vulnerable population) and, in addition, shows a propensity to evolve into more serious complications (dental pain, infections). A narrative review was carried out to support a protocol for treating ECC with efficacious and safe (in terms of SARS-CoV-2 transmission) procedures. Protocol involves criteria for patients’ selection remotely (telemedicine), and well-detailed criteria/equipment and hygiene procedures to combat against SARS-CoV-2 transmission. Moreover, the protocol proposes innovative caries treatments, named Minimally Invasive Treatments (MITs), well known in pedodontics for their high level of children’s acceptance during dental care. MITs allow for caries removal (particularly in primary teeth) without any high-speed rotating instrument cooled with nebulized air-water spray (with high risk of virus environmental diffusion), usually adopted during traditional treatments. For evaluating MITs effectiveness in caries management, only Systematic Review and Randomized Controlled Trials (RCTs) were included in our study, without any risk of bias assessment. The indications proposed in this protocol could support clinicians for the temporary management of ECC until the SARS-CoV-2 pandemic ends.

## 1. Introduction

### 1.1. The SARS-CoV-2 Pandemic and Its Social Consequences

SARS-CoV-2 infection represents one of the major medical emergencies in recent years, so much so that on January 30, 2020, WHO defined the virus pandemic as a public health emergency of international importance [1]. The rapid spread of the virus has led the governments of many nations to adopt interventions for the progressive containment of public social relations (“social distancing”) in order to reduce the sources of interpersonal contagion. This strategy led to the progressive closure or suspension of most of the community activities relating to the economic, social, cultural, and health fields in each country involved in the pandemic [2,3].

Public and private health services, capable of protecting the citizens from illnesses, have been forced to a sudden halt in activities in order to limit the risk of contagion from SARS-CoV-2. The management of oral health, including that of children, is part of this planned drop in health care services provided, to the detriment of correct daily care of the oral cavity [4]. This becomes more serious when vulnerable classes (physically and economically) are involved. For example, with children belonging to the socio-economically most disadvantaged classes and those with special needs, a reduced provision of dental care (especially public care) leads to a worsening of oral health status more marked than in any other social context where oral diseases are less prevalent [5,6]. This leads to an increase in the degree of inequality related to the right to health protection and equal access to fundamental care [7].

For this reason, in the present study, we are proposing a safe protocol (in terms of SARS-CoV-2 contamination), which at the same time guarantees minimum standards of care related to one of the most common non-communicable chronic diseases of the developmental age, Early Childhood Caries.

### 1.2. Peculiarities of the SARS-CoV-2 Infection in Children

Subjects of any age might be susceptible to infection, even if the people most affected (in terms of prevalence and severity of the disease) are those of more advanced age and who have concomitant chronic diseases [8]. At the moment, in fact, several international studies have reported that the majority of pediatric cases with COVID-19 demonstrate a reduced severity of the disease and no associated mortality events. Infected children can be asymptomatic, or have fever, cough, and fatigue as the most frequent symptoms. In some cases, gastrointestinal symptoms such as nausea, vomiting, abdominal pain, and diarrhea are associated [9]. A reduced percentage of children (around 5%), significantly lower than in adults, have dyspnea, and an even lower percentage (0.6%) develop into a more severe acute respiratory distress syndrome [10].

### 1.3. Transmission of SARS-CoV-2

The common transmission routes of SARS-CoV-2 are of two types: direct and indirect. Direct transmission occurs by inhalation of volatile droplets emitted by individuals infected with the virus when they speak, sneeze, cough, or breathe. The fact that the virus is harbored in both the lower and upper airways and in nasal secretions constitutes an additional element of risk [11]. In dental practices, the air-water spray, nebulized in a fine cloud of micro-droplets, produced by the drills (turbines and high-speed electrically powered micro-motors) during their use in the patient’s mouth, constitutes a very important additional vehicle for the spread of the virus in the environment [12]. Indirect transmission is also referred to as “by contact.” It occurs through physical contact between contaminated surfaces (hands, food, or objects) and the oral, nasal, and ocular mucous membranes of the subjects exposed to the virus [13,14,15].

### 1.4. Urgent and Emergency Dental Interventions

The international scientific community and the ministries of health of most countries involved in the SARS-CoV-2 pandemic recommend limiting treatments only to the urgent or emergency interventions. This is to avoid the spread of SARS-CoV-2 from patient to healthcare professionals and vice versa. Dentistry is one of the most at risk medical disciplines, as the oral cavity is part of the upper respiratory tract [16,17]. The most common emergency oral cavity interventions are for the management of pain (e.g., dental pulpitis, interdental gingivitis), and when there are localized or diffuse oral infections with the risk of reducing the lumen of the upper airways and aggravating the respiratory syndrome linked to SARS-CoV-2 [18]. Further urgent conditions are serious patient discomfort related to oral functions such as impaired chewing due to severe and numerous caries. For a more complete description, all oral emergencies and urgent clinical conditions for which dental services treatment must be guaranteed, also in the case of the SARS-CoV-2 epidemic, are listed in Table 1.

The recommendations described apply to adults as well as to children. Therefore, Early Childhood Caries, especially when severe, is a condition that cannot be postponed over time. This serves to prevent the evolution of caries into more serious and complex complications to be managed also in terms of protection from SARS-CoV-2.

### 1.5. Early Childhood Caries (ECC)

The American Academy of Pediatric Dentistry defines Early Childhood Caries (ECC) as the presence of at least one carious lesion (cavitated or non-cavitated) or a tooth extracted or filled for caries in one or more deciduous teeth in children up to the age of 71 months [19]. It represents one of the most common chronic infectious diseases in the world, with very high social costs. Children present masticatory or spontaneous (pulpitis or abscesses) pain and difficulty in chewing and speaking, as well as psychological effects such as a reduction in personal self-esteem [20]. The ECC is polarized in the socio-economically disadvantaged sections of the population, constituting an indicator of social inequality. Severe forms of this disease are characterized by at least one caries in the first three years of life or, after 36 months, by a dmft caries index greater than 3 at three years, 4 at four years, and 5 at five years [19,20] [AAPD 2008].

## 2. Methods

### 2.1. Study Design

In this article, an analysis (narrative overview) of the most current scientific evidence has been carried out to validate an intervention protocol aimed at an appropriate health care for pediatric patients affected by ECC in a period of pandemic.

### 2.2. Studies Selection and Data Extraction

The search strategy of this narrative review was divided into two parts. The first part addressed the analysis of the transmission routes of SARS-CoV-2 and the procedures to prevent its transmission in the dental office, in order to protect pediatric patients and their parents/caregivers as well as the dental staff. In this part, the main criterion that guided the research strategy was to identify the most recent studies, published in the best known international journals, which dealt with the topic of SARS-CoV-2 during its pandemic. No limitations were adopted in the selection of studies with regard to the type of study design. The results of this research have been described as “general safety criteria” in paragraph 3.1 of the Results section.

The second part of the research strategy was focused on the analysis of the effectiveness in managing ECC using the “minimally invasive treatments” identified as appropriate in reducing the risk of SARS-CoV-2 transmission. In this part, only systematic reviews of prospective clinical trials or single Randomized Controlled Clinical Trials (RCTs) were selected to ensure the most compelling evidence in support of the protocol purposed in this study. In addition, some in vitro studies were consulted only to reinforce the data published in the clinical studies. The data found during this second literature search were included in the paragraph 3.2 of the Results section as “specific safety criteria.” The most relevant databases, such as Pubmed, Embase, and Web of Science, as well as Cochrane Oral Health’s Trials Register and Cochrane Central Register of Controlled Trials (CENTRAL; 2019) in the Cochrane Library, were consulted. Book chapters dealing with the abovementioned topics were also considered in this review. Records found in the literature and those that were assessed to be useful for our analysis were obtained as full texts and data were extracted. Two researchers performed the records selection, while another two carried out the data extraction. Disagreements between researchers were resolved through discussion.

### 2.3. Inclusion and Exclusion Criteria

Although, in this review, preference was given to documents, which, based on the study design, ensured a greater degree of evidence, no type of study design reporting data on the topic of interest was excluded. Moreover, protocols were also evaluated. Although, preferentially, studies dealing with pre-school children (aged up to 71 months) were selected, the age of the study’s participants was not an exclusion criterion. Only studies written in English were considered.

### 2.4. Outcomes

Primary outcomes: dental treatment safety for patients and parents/caregivers against SARS-CoV-2 transmission; dental treatment safety for dental staff operators against SARS-CoV-2 transmission; caries treatment effectiveness; Treatments’ side effects. Secondary outcomes: participant discomfort; treatment duration; cost-effectiveness.

### 2.5. Statistical Analysis

The effectiveness of caries treatments reported in the studies selected for this review was described with the following relative indices: Odds Ratio (OR), Risk Ratio (RR), or Prevented Ratio (PR) with a 95% confidence interval (CI) for dichotomous outcomes between the treatment and control groups, while when continuous data were described, the Mean Difference (MD) with a 95% CI was used.

### 2.6. Results Section Structuration

As the purpose of this study was to provide clinicians with indications in the treatment of ECC during the SARS-CoV-2 pandemic, the Results section was structured as a Protocol instead of a more traditional design. Outcomes listed in the Methods were reported also in the Results and described as points of the following Protocol.

## 3. Results (Protocol for ECC Management during the SARS-CoV-2 Pandemic)

The dental treatment of early childhood caries in a period of crisis (SARS-CoV-2 pandemic) consists of a series of interventions that must satisfy a dual purpose: to guarantee the maximum degree of safety by avoiding any type of infection transmitted by the saliva droplets of the patient to the health workers (and vice versa), and to bring the patient back to a state of oral well-being. The protocol written in this study is based on following “general and specific safety criteria.”

### 3.1. “General Safety Criteria” for Avoiding SARS-CoV-2 Contamination from Saliva Droplets

The general criteria are a series of procedures to select patients who need dental care and manage them (and their parents/caregivers) in the dental office in absolute safety with regard to avoiding SARS-CoV-2 transmission. Of course, dental practitioners and their staff are intimately involved in these safety procedures. The first criterion is to select pediatric patients remotely who need to be treated urgently. In this sense, telemedicine (due to its high degree of diagnostic accuracy) can be very useful in reducing the influx of patients to oral health service facilities. Through telemedicine, it is also possible to establish a dialogue with parents in order to provide them with useful information on oral hygiene and correct non-cariogenic nutrition [21,22]. Establishing an early and repeated therapeutic alliance with parents produces positive oral health outcomes and represents the first of the interventions that the dentist must perform [23,24].

During the telematic visit, the general health of the patient and of the parent/caregiver appointed to accompany the child to the dental office can also be checked. The verification of any signs and symptoms related to the SARS-CoV-2 infection will allow the dentist to evaluate the advisability of carrying out dental treatment or to direct the parents of pediatric patients to contact specific medical services specializing in the treatment of COVID-19.

#### 3.1.1. Reduction of Number of Patients Treated Daily

In order to correctly carry out all the pre and postoperative sanitization procedures and to avoid crowding of patients (and parents/caregivers) in the waiting room, it is recommended to reduce the total number of patients treated daily depending on the organization of each single dental office.

#### 3.1.2. First Evaluation of the Patient’s General Health

The first step to be carried out in the dentist‘s office (after telephone or telemedicine triage) is the patient’s evaluation. The clinician must be able to identify a patient with COVID-19. The measurement of body temperature should be the first monitoring element, followed by the administration of a questionnaire on possible contacts with people infected or residing in areas judged to be high risk of infection. If body temperature exceeds 37.3 °C (99.14 °F) and the patient answers questions affirmatively, dental treatment should be postponed [9,11].

#### 3.1.3. Hygienic and Protective Measures for Dental Practice Staff

Following the indications of the West China Hospital of Stomatology, a handwashing protocol was proposed twice before and three times after dental surgery. It has been shown that over time there is a certain degree of difficulty in maintaining this habit [11,25]. The office staff must absolutely wear disposable gloves, masks and gowns, respirators, goggles, and face protectors, as well as washing hands as indicated above, with each patient. The omission of even one of these criteria significantly increases the risk of contagion [17]. One review describing the protective equipment for preventing highly infectious diseases with regard to gloves (RR 0.22; 95% CI 0.15 to 0.31), masks (RR 0.33; 95% CI 0.14 to 0.80), and powered air-purifying respirators (RR 0.27; 95% CI 0.17 to 0.43), showed results that these equipment are very effective against the transmission of illnesses [26].

#### 3.1.4. Work Surface Management

Work surface management must be reprogrammed according to the protocols included in the following two guidelines published by the National Health Commission of the People’s Republic of China:

(a) Guideline for the Prevention and Control of Novel Coronavirus Pneumonia in Medical Institutes (http://www.nhc.gov.cn/yzygj/s7659/202001/b91fdab7c304431eb082d67847d27e14.shtml).

(b) Guideline for the Use of Medical Protective Equipment in the Prevention and Control of Novel Coronavirus-Pneumonia (http://www.nhc.gov.cn/yzygj/s7659/202001/e71c5de925a64eafbe1ce790debab5c6.shtml).

In these guidelines, the most relevant disinfection-sanitation advice might be summarized as follows. In order to disinfect the surfaces exposed to salivary droplets (dental devices or work environment), those surfaces might be cleaned with gauze soaked with products based on ethanol of at least 70% or 0.1% sodium hypochlorite. When possible, the dental instruments might also be directly soaked in the cited disinfectant solutions. In both cases, the instruments’ surfaces have to be exposed to the disinfectant products for at least one minute. Dental office rooms should be sanitized after each visit by opening doors and windows for up to one hour. In dental offices without external openings to receive fresh air, a ventilation system with a high-efficiency particulate air (HEPA) filtration should be used.

#### 3.1.5. Oral Rinses

Some mouthwashes containing substances capable of reducing the infectious load of SARS-CoV-2 are indicated in the literature, such as Citrox (based on a combination of natural bioflavinoids extracted from citrus) and Amphiphilic β-Cyclodextrin (a modified glucopyranoside antiviral molecule deriving from glucose). These two rinses proved effective in reducing the salivary SARS-CoV-2 load due to their oxidizing action against this virus [27,28]. Citrox rinse showed additionally the capability of reducing the SARS-CoV-2 growth by inhibiting coronaviral chymotrypsin-like protease activity, the enzyme necessary for its replication. These two products might also be used in combination, resulting as both effective against virus infection and safe for patients [28]. In children, however, especially those of younger age, an oral rinse is not easy to perform correctly [29]. The adoption of a gauze soaked in mouthwash to swab and disinfect oral surfaces could, therefore, be an alternative solution for younger, uncooperative children [30]. With the presence of SARS-CoV-2 infection in the upper airways, the Benzydamine hydrochloride rinse can be very useful also in clinical practice in order to reduce the phlogosis of mucous membranes in children [31].

#### 3.1.6. Rubber Dam Use

The use of the rubber dam is strongly recommended. Studies show that the dam reduces the presence of suspended particles in the work environment by 70% [32].

#### 3.1.7. High Speed Drills

The use of drills (both turbine or electric powered micro-motor handpieces) that generate sprays of water and air should be avoided (unless strictly necessary) during the pandemic period due to the risk of contamination [12], while the use of hand-instruments (e.g., excavator) is preferred. However, the use of high-rotation speed drills could be necessary in case of endodontic urgency related to deciduous dental elements where it is not possible to complete the opening of the pulp chamber by using hand-instruments. In this case, we recommend the use of high-rotation speed electrically powered micro-motors without using the spray of water and air (https://www.aegisdentalnetwork.com/id/special-issues/2014/10/ power-to-the-handpiece).

Similar to traditional techniques, even the most innovative ones in caries removal such as laser (used at ablative energies) or sonic and ultrasonic instruments—both using handpieces with air and water spray—are not recommended because they are possible vehicles of infection transmission [33,34].

### 3.2. “Specific Safety Criteria” for Avoiding SARS-CoV-2 Contamination From the Use of Air-Water Sprays

Specific safety criteria are those adopted by dental practitioners to remove dental caries as an alternative to the use of drills (turbine and/or high speed electric powered handpieces), which, with their nebulized air-water spray, represent a relevant SARS-CoV-2 vehicle of contamination. Many characteristics of such specific safety criteria are satisfied by a particular typology of caries treatment known as Minimally Invasive Treatments (MITs).

#### 3.2.1. Minimally Invasive Treatments (MITs)

MITs are a wide range of caries treatment interventions that aim at the maximum conservation of the tissues and the least psychological impact on the patient. Said interventions are on average inexpensive, easy to apply, and do not necessarily presuppose high technology environments in order to be supplied. Because of their characteristics, they have been widely adopted, especially in the treatment of ECC, in more disadvantaged socio-economic and cultural environments and with uncooperative patients, such as children with fear of the dentist or subjects with special needs [35,36,37,38]. In all the categories just mentioned, where caries is more prevalent than in the basic population [6], minimally invasive techniques is a valid healthcare response.

The use of minimally invasive interventions has multiple positive characteristics in the management of caries during the SARS-CoV-2 pandemic. The minimally invasive interventions, in fact, are rapid in their execution (reducing the exposure times of health care workers), and have a high ability to stop caries and remineralize dental tissues while avoiding the most serious complications (e.g., pulpitis, abscesses). Some of the minimally invasive techniques allow the reconstruction of the anatomy of the tooth if affected by cavitated lesions. The minimally invasive techniques also have the essential advantage of avoiding the use of dental drills (high speed turbines and electrically powered micro-motors), which produce sprays of water and nebulized air, and which constitute a primary source of virus spread in the operating environment [12].

#### 3.2.2. Classification of Minimally Invasive Treatments (MITs)

We will only talk about a few MITs (those that seem more suitable to the present topic), listing them according to their degree of invasiveness. In this sense, “non-invasive” treatments are defined as those that do not involve any removal of the decayed dental tissue; on the other hand, “micro-invasive” treatments are those that involve the removal (partial or total) of the softened tissues through a hand instrument (excavator). We also believe that there should be no rigid hierarchy in the choice of which minimally invasive intervention to adopt or adopt first, leaving that choice to the clinician based on the individual case.

#### 3.2.3. Non-Invasive Treatments

##### Use of Fluoride Varnishes

The most commonly used type is 5% sodium fluoride varnish (22,600 ppm fluoride). This is employed as a primary prevention tool but also as an intervention to stop early enamel caries. Varnishes, used as prophylactic tools, reduce the incidence of caries in children and adolescents with a prevention fraction of 43% in permanent teeth (95% CI: 30% to 57%, *p* < 0.0001) and 37% in deciduous teeth (95% CI: 24% to 51%; *p* < 0.0001) [39]. The same varnishes are also effective in stopping enamel caries with a degree of remineralization of 63.6% (95% CI: 36.0% to 91.2%, *p* < 0.001) relating to both permanent and deciduous teeth in children. They are more effective when applied once every 6 months rather than annually [40].

##### Use of Silver Diamine Fluoride (SDF)

These are commonly used at a concentration of 38% (44,800 ppm fluoride). In a recent review of the literature commissioned by the Organization for Caries Research of the European Federation of Conservative Dentistry, silver diamine fluoride has shown a high degree of effectiveness in stopping the caries of deciduous and permanent teeth, with a degree of tissue remineralization varying from 79% to 91% in a population of children up to nine years of age [41]. When applied every six months, the degree of remineralization does not drop below 80% [40]. Using this technique, caries become dark and do not evolve. In addition to promoting dental remineralization, this type of agent has an antibacterial effect, inhibiting specific collagenases (matrix metalloproteinases and cysteine cathepsins) responsible for the digestion of demineralized dentine [42,43].

##### Use of Casein Phosphopeptide—Amorphous Calcium Phosphate (CPP-ACP) Products

These are milk protein derivatives capable of carrying high concentration calcium and phosphate ions, which, depositing on the hard tissues of the tooth, favor their remineralization. CPP-ACP is commonly available as a paste and can be administered to children directly by their parents, once a day, by spreading the product on the teeth with a finger. In the literature, the CPP-ACP has shown a certain effectiveness in remineralizing enamel white spots (SMD −0.43, 95% CI: −0.79 to −0.07, *p* = 0.02) [44]. However, the best results in stopping caries are obtained by combining the use of CPP-ACP paste with the most common products containing fluoride (toothpastes, mouthwashes, and varnishes), thus increasing the effectiveness of the latter (SMD −21.02, 95% CI: −27.94 to −14.10, *p* < 0.01) in terms of remineralization [45].

##### Use of High Viscosity Glass Ionomer Cements (HVGIC) in the Prevention of Carious Lesions

Caries prophylaxis is one of the key elements of childhood dentistry. The sealants and fluoride varnishes administrations are the most adopted prophylaxis procedures in child dentistry. The glass ionomer cements used as sealants are a valuable resource for their rapid execution and effectiveness in terms of caries prevention, preferable to interventions that require more time in the dentist’s chair. The literature shows an equivalent efficacy of HVGIC compared to resin sealants after 48 months of observation, and it is slightly better after 64 months (RR 0.29; 95% CI: 0.09 to 0.95; *p* = 0.04), even if the level of evidence is low [46]. The sealing constitutes, with both resinous and HVGIC materials, a highly effective solution in reducing caries in permanent first molars of children ages 5–10 years over 24 months of observation (OR 0.12; 95% CI: 0.08 to 0.19) [47].

#### 3.2.4. Micro-Invasive Treatments

##### Interim Therapeutic Restoration (ITR)

This is a technique that requires only a slight removal of decayed dentine around the edges of the cavity of the carious lesion, leaving most of it intact. The cavity is then directly filled by HVGIC with high fluoride release. The aim is to stop the progression of caries and remineralize the dental tissues. After a certain period, the filling can be replaced or simply monitored over time. This technique is primarily used in patients with reduced compliance and/or with severe ECC, allowing the treatment of multiple carious lesions in the same session. Despite the advantages indicated, the ITR has a higher degree of detachment of fillings than traditional fillings [48].

##### Atraumatic Restorative Treatment (ART)

This technique involves the removal of the carious dentine with a hand instrument (excavator) and the subsequent filling of the cavity with materials with slow fluoride release (HVGIC or resin-modified glass ionomer cement (RMGIC)). These cavities made with hand instruments can also be filled with other materials (e.g., composite resins). Caries must be completely removed when they are diagnosed as superficial or of medium depth, while a small part of softened dentine can be left in the cavity bottom in the case of deep caries without any pulp symptomatology [37]. ART allows for restoration of the anatomical shape of the tooth, eliminating the discomfort/pain experienced by the patient during chewing. However, fillings made with ART present a greater degree of detachment compared to those realized with traditional techniques over 12–24 months (OR 1.60; 95% CI: 1.13 to 2.27) [49]. With regard to post treatment dental pulp complications, the ART and traditional treatments were comparable when deciduous teeth with medium-deep caries were treated [50]. HVGIC can also be used in combination with silver fluoride diamine application, exploiting the benefits of both treatments. The use of HVGIC with antibacterial additives, in the correct proportions, can increase the effectiveness in caries control without altering the mechanical proprieties of the cements [51].

##### Chemo-Mechanical Techniques

In this type of approach, the carious dentine is first chemically softened and then removed with hand instruments. Based on the chemical substance used, these techniques are divided into two categories: those based on sodium hypochlorite (Cariosolv) and those based on collagen-lithic enzymes (Papacarie) [52]. When Cariosolv was compared with traditional techniques on permanent and deciduous teeth, no differences were found in terms of the duration of the fillings and post-treatment complications (*p* = 0.50). However, chemo-mechanical techniques require a significantly longer work time than traditional ones (*p* < 0.01), a fact that could limit their use, given the need to reduce the exposure time of dental staff to patient and vice versa [53].

##### Use of Sub-Ablative Energy Lasers

The most recent literature offers indications on the usefulness of some types of lasers (without spray of air and water such as the diode laser) in reducing the bacterial load in the bottom of deep cavities where softened dentin is left [54]. Moreover, a recent literature review of in vitro studies supports the effectiveness of multiple lasers used at low sub-ablative energies (including diode) in strengthening the enamel tissues against caries [34].

##### Use of Preformed Crown (Hall Technique)

This technique consists of the application of a pre-formed stainless steel crown (there are multiple sizes) directly onto a deciduous tooth, without any dental preparation or caries removal. It is usually used on deciduous molars affected by extensive caries or dental hypoplasia. The Hall technique, similar to traditional techniques that require tooth preparation, provides a longevity of the restorations over time comparable to traditional fillings in the short period of 12 months. Moreover, this innovative technique performs slightly better in the long period (RR 0.18; 95% CI: 0.06 to 0.56) [55].

**Figure 1:**
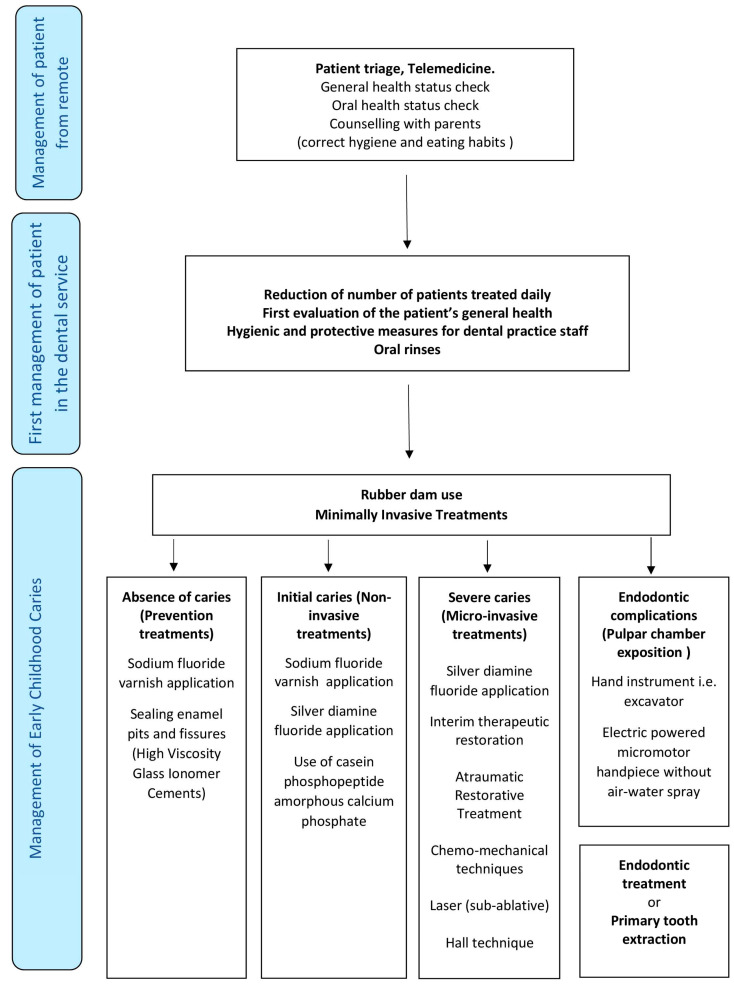
Flow Diagram describing the hierarchy of the procedures for the correct management of patients affected by Early Childhood Caries (ECC) during the SARS CoV-19 epidemic.

All the described criteria/procedures proposed in the present protocol for managing patients affected by ECC in a period SARS-CoV-2 have been schematized in the flow chart diagram drawn in Figure 1. Moreover, the hierarchy of all abovementioned procedures described in this paper is summarized in Table 2 in order to support dental staffs for adopting this proposed protocol in daily clinical practice.

## 4. Discussion

The proposed operative protocol refers specifically to the management of pre-school patients with Early Childhood Caries. Pre-school age is a very important period for oral health as it commonly coincides with the onset period of caries. Better managing of pre-school oral health means setting the child on a virtuous path that will protect him or her even in adulthood. Moreover, both the high prevalence and the rapid progression of ECC towards its infectious and painful complications motivate the choice for maintaining a continuous dental treatment regime even during the SARS-CoV-2 pandemic. For this reason, an appropriate protocol has been proposed to help solve this health emergency, which is valid during this pandemic. Otherwise, in the absence of ECC treatment, an increase in the burden of oral disease and the need for treatment are certainly expected after the pandemic with enhanced costs for public health as well as for families attending private dental facilities. In addition, the progression of ECC determines an increase in the number of dental extractions with a consequent impaired chewing function and an increased risk of malocclusions [56]. Malocclusions, in turn, constitute further social costs and the alteration of chewing function even in permanent dentition. Another element that aggravates the oral health condition of children is the sedentary life to which they are obliged during the SARS-CoV-2 pandemic. In this period, indeed, the interruption of ordinary relationship activities also involves children who remain inside their homes, favoring sedentary habits. A sedentary lifestyle appears to be related to poor eating habits that contribute to diseases such as obesity and caries [57].

Although this proposed protocol was designed to overcome the risk of ECC worsening during SARS-CoV-2, its usefulness in many respects could also be considered after this general period of crisis. Telemedicine, for instance, in addition to being an effective communication tool in the period of infectious risk from SARS-CoV-2, might well continue to be so even after, helping to establish a “therapeutic alliance” between the dentist and parents—an essential condition in children’s dentistry. Repeated counseling between specialists and families, even if done remotely, can in fact constitute a valid tool for motivating correct oral hygiene lifestyles. This method is also an excellent diagnostic and control tool for oral health, useful even in a condition of restored normalcy. In addition to telemedicine, even minimally invasive techniques, adopted for infectious risk, constitute a clinical resource regardless of the pandemic. In fact, these techniques are well accepted by children and promote their “compliance” with dental care, also contributing to the management of one of the most evident problems in childhood, namely the fear of the dentist. Regarding this type of childhood fear, telemedicine can also play a positive role by promoting a progressive familiarization of the child with the dentist and the adoption of well-coded psychological techniques to be continued in the dentist’s chair [58]. During the pandemic period, the use of simple and quick treatments, such as the application of fluoride gels and varnishes or the ART and ITR caries removal techniques, are also useful to compensate for the increased time required for the more complex disinfection and sanitization procedures used against the SARS-CoV transmission. Furthermore, the minimally invasive techniques, due to the absence of aerosols from high-speed handpieces, help reduce sanitization time in the dental operating room.

In recent months, several studies were found in the literature that related SARS-CoV-2 infection to the practice of dentistry. Most of them have formulated criteria to be adopted at dental offices, which are, in many aspects, similar to those listed in the “general safety criteria” of the protocol proposed in this study. These criteria can be summarized as follows: initial screening of the patient suffering from SARS-CoV-2, the identification of urgent clinical conditions, the adoption of procedures to protect both the dental staff and the patient, and the disinfection and sanitization of the work environment and dental instruments/devices. Further criteria included the disinfection of the mouth with antiviral mouthwashes, the use of the rubber dam, and the use of rotating instruments only when strictly necessary as, for example, in the case of endodontic complications. During the use of rotating instruments, clinicians were also advised to use reduced air-water sprays, double surgical aspirators, and only high-speed handpieces equipped with an anti-reflux device [11,59,60,61,62,63,64]. The aforementioned papers, unlike the present study, did not propose any alternative clinical solution to traditional caries removal techniques, such as minimally invasive treatments. Three additional studies reviewed noted the usefulness of telemedicine in the management of oral health conditions (e.g., caries or orthodontic treatments), thus proposing an innovative method of consulting with the patient [65,66,67,68,69]. The latter two of these studies considered the idea of using minimally invasive techniques as a viable and alternative clinical solution to traditional caries treatments for children because of their dangerous aerosols [70,71]. These studies offered a critical analysis on the possibility of adopting minimally invasive techniques, while our protocol constitutes a real point by point guide for the pediatric dentist in treating ECC with total respect for the safety criteria related to the SARS-CoV-2 infection. Most precisely, the idea of supporting the pediatric dentist with an exemplified guide (based on the best scientific evidence) is the principal aim of our study. Moreover, one of the studies just cited [71] highlights the high percentage of children with SARS-CoV-2 having only mild symptoms or without symptoms, who risk being considered healthy and, therefore, not requiring specific care-taking protocols such as those indicated in this study.

### Study Limitations

The general crisis condition in which we find ourselves requires the immediate implementation of an ECC treatment protocol. This short time frame led us to write only a narrative (instead of systemic) review of the scientific literature supporting the aforementioned protocol. In addition, no detailed quality assessment of the studies included in this reviefw (involving a risk of bias evaluation) was possible. The only guarantee of quality was the choice (at least in the Minimally Invasive Caries treatment topic) of articles with a valid study design: systematic reviews and RCTs. In addition, the protocol proposed in this study is only useful for pre-school tooth decay without considering this disease in older patients. However, this first protocol could be a stimulus for other similar documents focused on caries treatment even in adulthood through safe procedures in terms of SARS-CoV-2 transmission.

## 5. Conclusions

ECC is a worldwide health problem. The suspension of dental health treatments during the SARS-CoV-2 pandemic could well affect ECC status in pre-school children, particularly in the socio-economically disadvantaged groups of populations where the disease has a higher degree of prevalence and severity. A new protocol for taking care of patients with ECC has been proposed based on the most current scientific evidence—one capable of continuing to provide dental care to children of pre-school age in a safe, low risk of contagion from SARS-CoV-2 environment. In fact, although children affected by SARS-CoV-2 demonstrate an evident symptomatology more rarely than do adults, they nonetheless can be carriers and transmitters of the viruses.

This protocol supports clinicians as a “secondary prevention” against ECC aggravation and its complications. Prevention is always the best strategy in children’s dentistry, in terms of health gain, reduction of costs, and social inequity, particularly for the benefit of the categories of vulnerable subjects who, in times of global crisis, could increasingly be excluded from future public health policies based on the protection of the population groups with a greater chance of overcoming the state of general calamity.

In addition, when the most important phase of the pandemic ends, everything will not return immediately to as it was before. It will be necessary to start again gradually, and protocols such as the one we have proposed for the care of children might be useful for another as yet undefined time in the future. In summary, after the lockdown phase, a period begins in which dentists are asked to balance the risks, particularly for children (maximizing the safety procedures against SARS-CoV-2 transmission), and the need to satisfy oral health requests [72].

## Figures and Tables

**Table 1:** The table shows the emergencies and urgent dental treatments that must be delivered even in a SARS CoV-2 pandemic, recommended by the American Dental Association.

Dental Emergencies
Trauma involving facial bones
Tooth or mandibular fracture
Pericoronitis or third molar pain
Incomplete tooth extractions
Surgical post-operative osteitis
Uncontrolled bleeding
Dental treatment required prior to critical medical procedures
Abscess
Infection with intra-oral or extra-oral swelling that potentially compromise airway patient
Severe resistant dental pain from pulp inflammation
Cellulitis or a diffuse soft tissue bacterial infection
Additional urgent dental treatments
Extensive dental caries or defective tooth development
Restorations causing pain
Suture removal
Denture adjustment on radiation/oncology patients or when function impeded
Replacing temporary filling
Endodontic treatment for patients experiencing pain
Biopsy of abnormal tissue

**Table 2:** The table shows the point to point workflow form management of a patient with ECC in the SARS-CoV-2 pandemic.

Hierarchy of Procedures	Type of Procedures		Description of Procedures
From remote	Telemedicine		Child has to be visited by dentist from remotely through the use of a phone video camera. The child’s general health status has to be evaluated as well. In this step, the dentist decides if a visit with the patient in the dental office is indicated or if the patient can be managed remotely.
	Scheduling dental office appointments		It is necessary to avoid crowding in the waiting room. Therefore, the number of appointments must be reduced to allow the sanitization of the working environments and the disinfection/sterilization of the dental instruments between two consecutive patients.
Dental office: waiting and service rooms	Before visiting the patient		The dental office staff has to be completely protected with gowns, disposable gloves and mask, and powered air-purifying respirators. The eyes should be protected with goggles or total face protectors. Before donning protective gloves, it is necessary to wash one’s hands twice, and after their use, three times again.
	Triage of patient and parents		As a first step in the dental office, the temperature of both child and his parent/caregiver must be taken and must measure lower than 37.3 °; a questionnaire about their general health status in relation to the SARS-CoV-2 pandemic must be filled out.
Dental office: dental chair room	Oral cavity disinfection		At the beginning of the visit, the patient must rinse for one minute with a solution of 0.5–1% oxygen peroxide or with iodine-povidone diluted 0.23% for at least 15 s.
	Teeth isolation		Rubber dam can be used to prevent the spread of saliva droplets from the patient’s mouth.
	Minimally Invasive Treatments	Caries prevention	Educative intervention on correct oral health habits. Use of sodium fluoride varnishes or High Viscosity Glass Ionomer cements (HVGIC) sealants for prophylactic interventions.
		Enamel caries	Use of sodium fluoride gels/vanishes or silver diamine fluoride (SDF), or prescription of casein phosphopeptide—amorphous calcium phosphate for home use.
		Dentine caries	Use of silver diamine fluoride (SDF), atraumatic restorative treatment (ART), interim therapeutic treatment (ITR), Chemo-mechanical techniques, sub-ablative laser, or Hall technique.
	Dental complication treatments	Pulpits or abscesses	Opening primary tooth pulpal chamber with excavator or with burs powered by electric micromotor without water-air spray. In addition, endodontic treatment or primary tooth extraction.
Dental office: after the visit	Sterilization, disinfection, and sanitation		Disinfection of non-autoclavable instruments/devices with a gauze soaked with ethanol solution of at least 70% or 0.1% sodium hypochlorite. When possible, the dental instruments have been directly soaked in the aforementioned solutions. The devices/instrument surfaces must be exposed to the disinfectants for at least one minute. Correct sanitation of work environment by opening doors and windows after each visit for up to one hour. In case of absence of external openings, the environmental sanitation might be made using a high-efficiency particulate air (HEPA) ventilation system.
